# Antibiotic-impregnated cement spacer as a definitive treatment for post-arthroscopy shoulder destructive osteomyelitis: case report and review of literature

**DOI:** 10.1007/s11751-013-0176-5

**Published:** 2013-09-12

**Authors:** Sleiman Haddad, Pablo S. Corona, Maria M. Reverté, Carles Amat, Xavier Flores

**Affiliations:** 1 Department of Orthopedic Surgery, Septic and Reconstructive Surgery Unit, University Hospital of Vall d’Hebron, Passeig de la Vall d’Hebron 119-129, 08035 Barcelona, Catalonia, Spain; 2 Universitat Autonoma de Barcelona, Barcelona, Spain

**Keywords:** Shoulder, Arthroscopy, Destructive, Infection, Cement, Spacer

## Abstract

Arthroscopic revision of rotator cuff lesions is an increasingly popular procedure with a relatively safe profile. However, associated deep articular infection has been described, with potentially destructive joint sequelae. When occurring, it poses the double challenge of eradicating the infectious agent while preserving the articulation and its function. Experience remains scarce and is mostly based on case reports and small series. These also rely on the evidence from the better-described lower extremity joint infections. Through a complex case, the following report addresses this exceptional situation and offers an unusual solution, taking into consideration the peculiarities of the shoulder joint. With the consent of the patient, a single-stage resection arthroplasty with the implantation of an antibiotic-impregnated cement spacer was performed as a long-lasting—if not definite—treatment. After 4 years, the patient maintains excellent function with no radiological signs of wear or loosening.

## Introduction

Arthroscopic revision of rotator cuff lesions is an increasingly popular procedure nowadays due to the convenience of its minimally invasive nature, associated with a relatively safe profile [1, 2]. One of the most feared complications of shoulder arthroscopy, although rare, remains deep articular infection, with potentially destructive joint sequelae [2–4]. When occurring, it poses the double challenge of eradicating the infectious agent while preserving the articulation and its function [2, 3, 5]. Experience with this rare occurrence remains extremely scarce and is mostly based on case reports and small series, reflecting experience with the more common post-arthroplasty infections [6]. These also rely on the evidence from the better-described lower extremity joint infections. The following report addresses this exceptional situation and offers a curious and an uncommon solution taking into consideration the peculiarities of the shoulder joint.

## Patient

We present the case of a 71-year-old woman referred to our outpatients department with an actively draining fistula on her right shoulder.

**Fig. 1:**
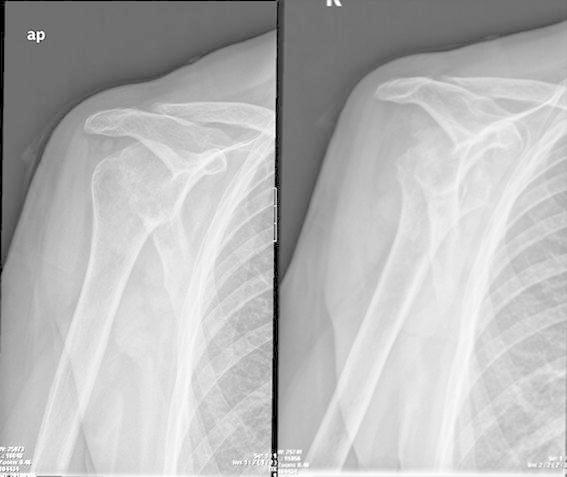
Shoulder X-ray revealing an irregular humeral head, with a bone defect on its greater tuberosity, which could correspond to the previous suture insertion

**Fig. 2:**
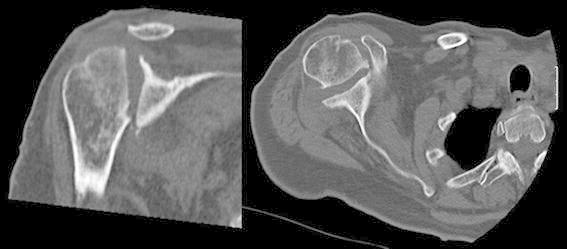
CT-scan images showing marked glenohumeral destruction, humeral head flattening, and articular narrowing

**Fig. 3:**
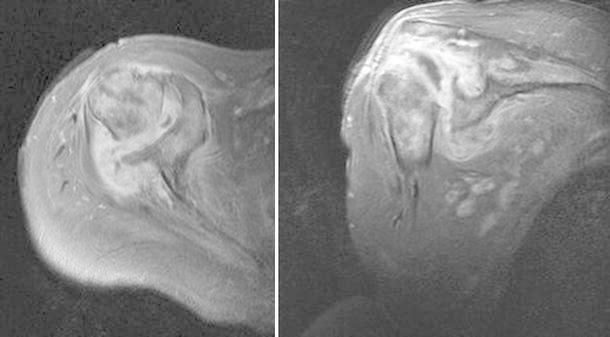
MRI imaging showing synovial inflammatory proliferation as well as contrast-enhancing joint effusion and chronic and active osteomyelitis of the humeral head and scapula. The rotator cuff muscles showed marked inflammatory changes and atrophy, and the supraspinatus muscle had lost its continuity

Her present history goes back to nearly 18 months prior to presentation when she underwent an arthroscopic rotator cuff repair with anchor suture of the supraspinatus muscle, at an outside center. In her immediate post-operative course, she explains a wound infection that necessitated an arthroscopic debridement and antibiotic therapy. Upon failure, she had an open articular lavage and debridement, with no avail. And finally, 9 months before her visit, she was submitted to another open revision and anchor suture removal. She failed to provide detailed reports and bacterial cultures for further studies. At the time of her first visit to our clinics, the patient was afebrile, with a preserved general state. Shoulder examination was difficult due to extreme pain, and anteriorly, along the surgical scar, we could observe a 1-cm sinus tract. Analytical workup showed no significant leukocytosis or inflammatory markers, except for an ESR of 53 mm/h. Shoulder X-ray revealed an irregular humeral head, with a bone defect on its greater tuberosity, which could correspond to the previous suture insertion (Fig. 1). Also, the deltoid muscle shadow was effaced, in accordance with a generalized local muscular atrophy. A subsequent CT-scan confirmed a marked glenohumeral joint destruction, with a loss of the spherical humeral head morphology, a narrowed articular space, and a flattened articular surface. We could also detect irregular trabecular morphology of the humeral head and some marginal proliferative changes (Fig. 2). Magnetic imagery demonstrated an important and generalized synovial inflammatory proliferation as well as contrast-enhancing joint effusion. There was an alteration of the bone signal of the scapula and most importantly, of the proximal humerus, suggestive of chronic and active osteomyelitis. The rotator cuff muscles showed marked inflammatory changes and atrophy, and the supraspinatus muscle had lost its continuity (Fig. 3).

Decision was made, with the consent of the patient, to perform a resection arthroplasty of the humeral head with the use of antibiotic-impregnated cement spacer as a prolonged—if not definitive—functional prosthesis.

A longitudinal trans-deltoid surgical incision was made, conditioned by the previous surgical scars, taking away the fibrous tissue and the fistulous tract. During the surgical approach, we could observe a severely thinned deltoid muscle, with grossly evidenced atrophic and inflammatory changes. The synovial fluid was replaced with a fibrous and purulent magma that was extensively washed after taking samples for cultures. The rotator cuff was nearly inexistent, with completely deficient supraspinatus and subscapular muscles and a much thinned infraspinatus muscle. The long head of the biceps was avulsed proximally from its origin on the glenoid fossa. Compared with the CT images, we could verify a severe joint involvement, with a humeral effacement. The glenoid fossa was better preserved than was originally thought. We then proceeded to a deep articular debridement. A 45° humeral head osteotomy was performed, and the medullar canal was prepared for the insertion of the humeral Polymethyl Methacrylate (PMMA) gentamicin-impregnated articulated spacer (manufactured by Tecres S.p.a. Sommacampagna (Verona) Italy). We reinforced the spacer with a gentamicin cement collar (Refobacin^®^ Bone Cement manufactured by Biomet (Indiana) USA) enriched with vancomycin. The use of the cement collar was double. First, we wanted to insure a higher spacer stability and durability. Second, we aimed at a broader coverage against methicillin-resistant Staphylococcus aureus (MRSA). Articular congruence was verified before wound closure.

## Results

Surgical cultures grew a nonresistant Staphylococcus aureus, and the patient was started on a three-month suppressive antibiotic therapy according to our hospital protocols. The post-operative course was uneventful with an adequate evolution of the surgical wound.

At the one-year visit, the patient was pain-free and had regained a satisfactory range of motion, with no restrains in her activities of daily living. She even regained full competitiveness in her recreational practice of Pétanque. She had a 60° active forward flexion, 70° abduction, 30° external rotation, and an internal rotation reaching T10 (vs. T5 in the contralateral arm), all of which were comparable to those reported in the literature in similar cases[7–9].

Functionally, she achieved a score of 70 on the American Shoulder and Elbow Surgeons (ASES) Standardized Shoulder Assessment Form (maximum score of 100) and had a Simple Shoulder Test score of 5 (maximum score of 12), also comparable to reported series [7, 10]. Her Quick Disability of the Arm, Shoulder, and Hand score (Quick-DASH; range 0–100 reflecting disability) [11] improved from 20.90 (at 6 months) to 15.90 at last control, 4 years after the index surgery, even better than described in other articles, in patients receiving two-stage exchange arthroplasty [7, 8].

**Fig. 4:**
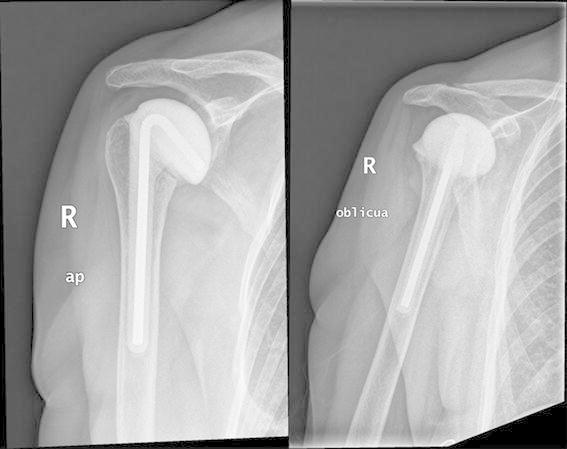
AP and oblique views of the shoulder joint at the 4-year follow-up showing no evidence of spacer loosening, secondary displacement, or glenoid wear

The patient’s inflammatory markers had normalized at 2 months after surgery, and the last radiological follow-up revealed no spacer loosening, secondary displacement, or glenoid wear (Fig. 4).

## Discussion

Shoulder arthroscopy is a relatively safe and minimally invasive orthopedic procedure, offering good functional results [1]. It has grown in popularity over the past decades specially basing on these highly praised advantages. However safe, retrospective studies have been reporting a slightly increasing complication rate, reaching between 4.8 and 10.6 % [2, 3]. Infectious complications remain most dreaded, affecting between 0.04 and 3.9 % [2], most of them superficial. Deep infections occur less frequently, affecting between 0.27 and 1.94 % of patients [4], and require multiple surgical debridements, ranging between 2.6 and 3.3 [4, 6]. Deep osteomyelitis after rotator cuff injury is even scarcer, accounting for only one case of the 25 described in a large series [4]. As such, destructive osteoarticular infection of the shoulder, posterior to shoulder arthroscopy procedures, is highly exceptional and challenging. Experience with such cases is generally based on the more common shoulder post-arthroplasty osteomyelitis and is reserved to specialized referral centers. As in periprosthetic joint infection of the shoulder, it can lead to joint destruction, extreme pain, and severe functional impairment.

Superficial wound infections after arthroscopy are successfully treated using topical wound measures and antibiotic regimens. These antibiotics should target common skin flora (including methicillin-resistant Staphylococcus aureus depending on local prevalence). Infection with *Propionibacterium acnes* has been gaining importance [4, 12] and should be suspected and not excluded until after 14 days of culture [12].

*Propionibacterium acnes* resistance is increasingly being reported nowadays, secondary to wide use of antibiotics for acne vulgaris, especially tetracyclins [13]. Adding an NSAID may help limit articular cartilage destruction in non-prosthetic infections [14].

However, many superficial infections of the shoulder may be just hiding a deeper joint involvement. Successful treatment of deep infections is based on correctly identifying the pathogenic organism, surgical debridement, antibiotic treatment, and repair or reconstruction of the soft tissue envelope [15]. Proper identification of the responsible pathogen and its susceptibility profile is of paramount importance to the success of any antibiotic regimen. Ideally, suture anchors and infected suture materials should be removed [15]. Re-repair of the rotator cuff or deltoid muscle with absorbable, non-braided suture material should be tried. If the quality of the tendon is compromised, it may be indicated to consider a muscular flap transfer [15, 16]. Literature, however, reports very few cases of deep infection after arthroscopic shoulder surgery, and no proper guideline can be drawn [4, 15, 17]. All of the reported cases were acute and accordingly debrided arthroscopically or through a combination of open and arthroscopic approach in more advanced cases. Our case stands out as a chronic infection where repeated debridement failed to eradicate the infection and only managed to amplify the local destruction and deep bone involvement. Failing to find similar cases, we considered our patient candidate to a resection arthroplasty.

As such, consensus on the best therapeutic approach to deep shoulder infection is still to be found, and our practice is based on the experience with shoulder arthroplasty [9, 15, 18, 19]. This latter is also still poorly defined and extrapolated from the highly documented and referenced experience with destructive articular infections of the hip and knee [9, 18, 20–22]. In this field, the standard procedure is a two-stage exchange, which could achieve the highest eradication rate and better functional outcome [21, 22]. So far, many authors have reported the successful implementation of this strategy to the infected shoulder prostheses [18, 19, 23, 24]. Other treatment options include chronic antibiotic suppression, debridement with implant retention, resection arthroplasty, 1- or 2-stage exchange, arthrodesis, and amputation. Antibiotic therapy alone or combined with debridement and implant retention had recurrent infection in up to 60 % of the cases and is now reserved for selected cases with acute infection only [19, 25]. Resection arthroplasty resulted in poor pain control and function in nearly 50 % of cases and a high infection recurrence [25, 26]. Also, resection arthroplasty with no spacer placement may impede a future reimplantation of prosthesis due to rotator cuff muscle disuse atrophy, soft tissue contractions, and disuse osteopenia of the humeral head and stem [27].

The introduction of the spacer to the first-stage resection of shoulder arthroplasty is relatively new as said and was based on the experience of the lower extremity [7–10, 19, 24, 28–32]. It combines the biomechanical advantages of retaining soft tissue tension, preventing atrophy and retraction, as well as offering an articulated surface to those of procuring antibiotic features locally with long-release formulae. As such, the antibiotic-impregnated articulated spacer becomes an important adjuvant in serving our three primary goals: eradicate the infection, relief pain, and provide articular functionality.

The natural evolution of this spacer passed through the artisanal form of an intraoperative manually molded spacer [9, 32] to use prefabricated molds during the surgical act [7, 8]. The latter offered the advantages of standardized modularity, to those obtained by a larger stem and a more regular articular surface. It had the inconvenience of consuming precious intraoperative time. The last product to come into use is a commercially produced antibiotic-impregnated cement spacer [10], the same used in our patient. It is also advocated to deliver a constant therapeutic antibiotic concentration locally over a period of 99 days [33]. To date, our patient is the first to benefit from this spacer, outside of the series by Coffey et al. This spacer was originally thought to be an intermediate step into the final arthroplasty in a two-stage procedure. The original product description sheet by Tecres recommended the spacer to be used as a temporary prosthesis for a period not exceeding 180 days. This was surpassed in our patient, as well as in the series by Coffey et al. On successive follow-up visits, the patient did not lose functionality, or complain of pain, and furthermore, her quick-DASH score improved over that recorded at 6 months. One explanation might be that the extrapolation from the lower extremity experience is not completely accurate. In fact, the shoulder, being a low impact articulation with no axial charges, is less demanding than the hip or knee, better tolerates, and erodes less the cement implant. Therefore, the largest follow-up on a patient treated with manually crafted spacer was up to 5 years with satisfactory results [32]. Other series report high implant longevity, in patients refusing the second surgical act, with a last follow-up between 20 and 28 months [7, 8, 10]. Also, Stine et al. [7] observed no intraoperative gross glenoid wear in patients opting for prolonged spacer implantation on the latter arthroplasty revision. However true, little is known about the cement–bone articulating surface, its degeneration, or its long-term results. As such, this option has been elected by the patients at the time of the second stage, not introduced by the surgeon at the time of the first interview, and was reserved to compromised patients. Contrary to other studies, our patient was explained the literature evidence at the time of the first encounter and opted voluntarily for the spacer as a prolonged/definitive solution. This decision was even more reinforced with the recent evidence from the latest series. No statistical evidence was found between the two-stage arthroplasty and the resection arthroplasty with the cement spacer in both functionality and infection eradication [10].

The presented case is the first case of post-arthroscopy shoulder infection treated with extended if not definitive antibiotic-loaded cement spacer. Our patient remains free of infection at the last control, based on her inflammatory marker and the clinical control. She has a comparable range of motion when compared to the infected arthroplasty population, receiving a two-stage exchange arthroplasty, and better functional scores than this same population. The last radiological control at 4 years shows no spacer loosening or joint osteolysis. Furthermore, the patient is completely satisfied with her primary decision of receiving a long-lasting or definitive spacer. The presented patient was the first in a series of three, with the second patient just crossing the 2-year mark.

Based on our experience, we recommend that orthopedic surgeons actively explain and offer the alternative of a prolonged/definitive implantation of the antibiotic-impregnated cement spacer, to patients suffering from deep destructive shoulder infections, at the time of first encounter. This option is gaining in evidence, and when failing, does not impede proceeding to the current accepted standard of care in destructive joint infections. We recognize that further research is still needed to study the cement–bone articulating surface both in vitro and in vivo, taking into consideration the mechanical peculiarities of an articulation with low axial loads such as the shoulder. These might help change the treatment paradigm in deep shoulder infections saving yet another surgical aggression to a repeatedly violated joint, in a compromised patient.
